# EndoTrainer: a novel hybrid training platform for endoscopic surgery

**DOI:** 10.1007/s11548-023-02837-x

**Published:** 2023-02-13

**Authors:** Albert Hernansanz, Ramon Rovira, Joan Basomba, Roger Comas, Alícia Casals

**Affiliations:** 1 Research Centre for Biomedical Engineering. (CREB), Technical University of Catalonia (UPC), Jordi Girona 1-3, 08034 Barcelona, Catalonia Spain; 2 Gynaecology and Obstetrics dept., Hospital de la Santa Creu i Sant Pau, Sant Quintí, 89, 08041 Barcelona, Catalonia Spain; 3 SurgiTrainer, Jordi Girona 1-3, Omega Building Off. 408, 08034 Barcelona, Catalonia Spain

**Keywords:** Hybrid surgical training platform, Endoscopic training, Endoscopic assessment, Surgical simulation

## Abstract

**Purpose:**

Endoscopy implies high demanding procedures, and their practice requires structured formation curricula supported by adequate training platforms. Physical platforms are the most standardised solution for surgical training, but over the last few years, virtual platforms have been progressively introduced. This research work presents a new hybrid, physic-virtual, endoscopic training platform that exploits the benefits of the two kind of platforms combining realistic tools and phantoms together with the capacity of measuring all relevant parameters along the execution of the exercises and of providing an objective assessment performance.

**Methods:**

The developed platform, EndoTrainer, has been designed to train and assess surgical skills in hysteroscopy and cystoscopy following a structured curricula. The initial development and validation is focused on hysteroscopic exercises proposed in the Gynaecological Endoscopic Surgical Education and Assessment (GESEA) Certification Programme from The Academy and European Society for Gynaecological Endoscopy (ESGE) and analyses the obtained results of an extensive study with 80 gynaecologists executing 30 trials of the standard 30 degree endoscope navigation exercise.

**Results:**

The experiments demonstrate the benefits of the presented hybrid platform. Multi-variable statistical analysis points out that all subjects have obtained statistically significant improvement in all relevant parameters: shorter and safer trajectories, improved 30-degree endoscope navigation, accurate positioning over the targets and reduction of the execution time.

**Conclusion:**

This paper presents a new hybrid approach for training, and evaluating whether it provides an objectivable improvement of camera navigation endoscopic basic skills. The obtained results demonstrate the initial hypothesis: all subjects have improved their camera handling and navigation skills.

**Supplementary Information:**

The online version contains supplementary material available at 10.1007/s11548-023-02837-x.

## Introduction

Natural orifice endoscopic procedures demand specific technical and cognitive skills as well as dexterous manual operation. Its performance is associated with certain challenges, due to the fulcrum effect, loss of binocular vision, limited haptic feedback and reduced mobility. These types of surgical techniques require specific education programs supported with realistic training platforms. In this respect, different medical associations are developing training and assessment programs (e.g. GESEA MIGS for gynaecologists and CME/CPD from EU-ACME for urologists).

Surgical training uses physical platforms, virtual simulators and to a lesser extent animal and cadaver models. Each approach has its advantages and limitations, varying their level of realism, evaluation and feedback capabilities, availability and price. All these methodologies require from mentors, a limited resource, in hands-on courses and continuous formation, that provide support to the mentees. From the analysis of training needs and the limitations of current training systems, a new hybrid physic-virtual approach, EndoTrainer (ET). ET is based on a physical platform with multiple modules reproducing the anatomies that uses real surgical tools and endowed with the capabilities of measuring, evaluation and interactivity usually provided by virtual platforms [1]. ET is introduced in this paper assessing basic surgical skills in hysteroscopy.

The paper is structured as follows. Section ‘Related work’ reviews current advances and trends in physical and virtual training platforms. Then, Sects. ‘Method’ and ‘Results’ describe the ET platform and the first two developed exercises for hysteroscopy. Following, Sect. ‘Discussion’ reviews the results obtained in a multi-center study to evaluate the 30 degree camera navigation exercise

## Related work

Animal models and cadavers played a predominant role in the past and are still an important training method nowadays. However, several problems prevent their extensive use: limited availability, high cost, differences from human anatomy (animals) and between live and dead anatomies and, finally, ethical issues. In fact, studies such as [2] relativise the effectiveness of training using cadavers. In this context, the use of training platforms emerges, staring from simple physical training setups and deriving to complex virtual simulators. Each platform offers complementary performances.

Evidence shows that the acquisition of surgical skills requires structured training programs supported by realistic platforms, [3, 4]. In [5], authors report hysterocopic skills training and assessment, pointing the low number of validity evidence studies. Modern trainers should allow objective assessment of the evolution of surgical skills and provide formative feedback, [6, 7].

### Physical training platforms

These training platforms are based on physical reproductions of the specific anatomy and use real surgical tools, achieving different degrees of visual and force feedback realism. However, the amount of exercises and critical situations reproduction is limited. In addition, they do not provide automatic and objective measures to quantify subjects’ performance, so they require a mentor to asses and to evaluate the quality of the executed exercises. Physical platforms are extensively used in laparoscopy and, to a lesser extent, in hysteroscopy or cystoscopy. Some physical platforms have been validated, most of them based on Objective Structured Assessment of Technical Skills (OSATS) [8]. Bozzini®Hysteroscopy provides a realistic reproduction of uterus with different pathologies. The used material can be resected with energy. Other examples are LYRA Hysteroscopy Trainer “EVA II” by Karl Storz, HYSTT training package from IDTrust Medica, [9], etc. None of them measure any parameter, neither evaluate the execution of the exercises. Several authors have proposed specifications of the more generic models of OSATS for hysteroscopy, [10].

### Virtual training platforms

Virtual simulators generate synthetic surgical environments using 3D modelling and rendering techniques to reproduce surgical scenarios, tools and their interaction. The benefits of this approach are diverse: first, the automatic and objective evaluation of exercises execution; second, a wide range of exercises, scenarios, surgical tools and critical events can be generated. However, in spite of the actual computing power, current simulators still lack of enough realism due to the accurate mathematical models required for physics simulation. In addition, virtual training platforms are still expensive and their massive introduction is not yet a reality.

Some platforms, like HYST Mentor, have already been validated [11]. The contribution and effectiveness of virtual trainers has been widely discussed, but there is a general consensus on the fact that their contribution is positive, [12]. Other researches conclude that virtual trainers are specially effective for novices, [13]. Automatic assessment is one of the main advantages of virtual simulators, [14]. Their validation is studied in [15], comparing expert assessment using specific OSATS with the automatic assessment and feedback provided by HystSim platform.

Haptic feedback plays a major role in acquiring surgical skills and increasing realism in training. Virtual platforms still require the development of consistent and robust haptic feedback systems, [16]. Virtual fixtures or cognitive simulation, including visual, audio and force feedback have demonstrated the benefits for training surgical skills in virtual simulators. Most of these studies are oriented to laparoscopy, [17–19].

### Hybrid training platform

Hybrid platforms combine both approaches to exploit their benefits and minimise their shortcomings. Hybrid physical/virtual platforms are based on a physical device that reproduces the anatomical cavity, allows the use of real surgical tools and presents the capability of measuring and evaluating in real time the execution of the exercises. In addition, they introduce Augmented Reality (AR) to generate interactive feedback to simulate different critical situations and act as a virtual mentor. Orthopaedic surgery, with rigid anatomical structures, facilitates the implementation of this type of simulators, [20, 21]. [22] presents an hybrid training platform for sentinel lymph node biopsy.

Concerning Minimally Invasive Surgery training, just a very few hybrid platforms like LTS3e laparascopic simulator have been validated, [23] or PRoMIS by Haptica. VirtaMed AG has a collection of virtual simulators with realistic physical setup to simulate ergonomics and realistic surgical conditions. Laparo Medical Simulators has developed a physical laparoscopy training platform that tracks and analyses the tools trajectory offering different evaluation metrics, but there are no sensors on the workspace to measure the interaction of the tools with the environment.

## Method

### EndoTrainer hybrid platform

The EndoTrainer platform follows the described hybrid approach: a) a physical platform containing the specific anatomy, real surgical tools, endoscopic camera and the required sensors to measure users actions; and b) the capability to evaluate users performance, generate personalised curricula based on their evolution and use AR to reproduce critical events and interactivity between platform and users.

Measurement and evaluation capacities are essential and represent the link between the physical and virtual platforms. Real-time data fusion provided by the Tool Tracker Arm (TTA), computer vision and sensors on the workspace opens the possibility of multi-parametric evaluation and the introduction of AR during the exercise execution. The capacity of real-time evaluation, augmented reality and user interactivity enables the serious gamification approach, generating personalised users’ curricula, proposing challenges to motivate their improvement and insight in those aspects in which the results do not correspond to the expected learning evolution. Multiple studies demonstrate the benefits of gamification in training, [24–26].

**Fig. 1:**
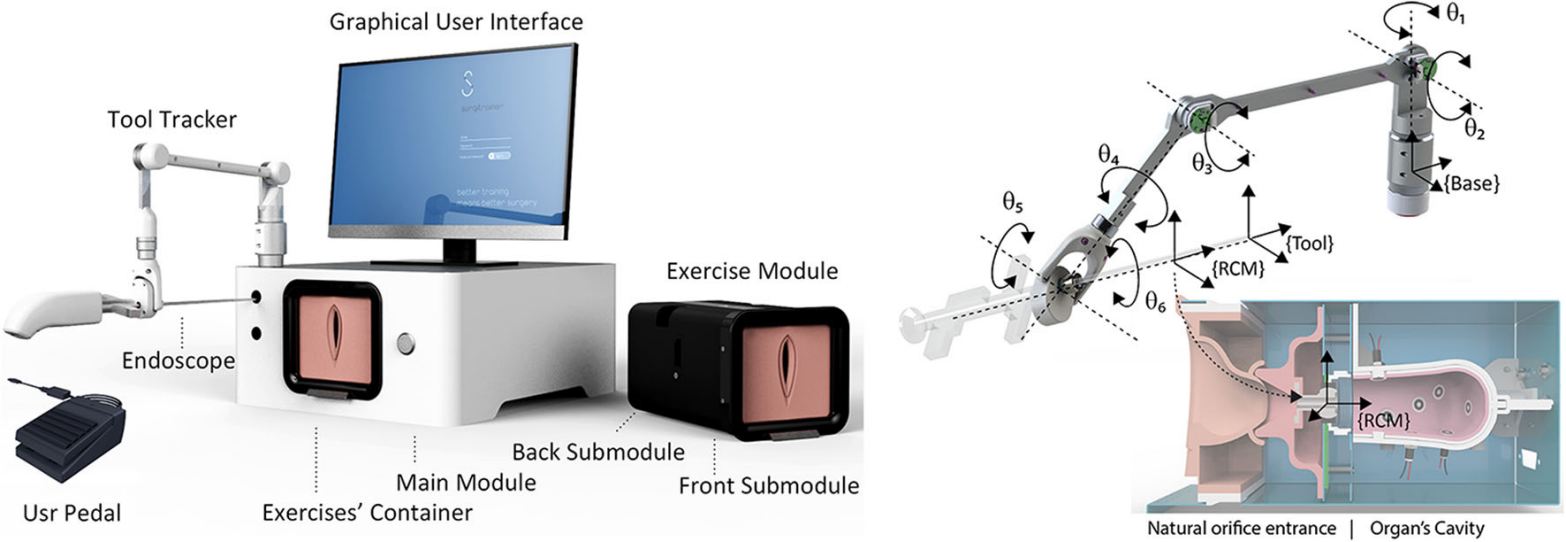
The ET components configured for hysteroscopic training (left). ToolTracker kinematics and illustrative rendering of exercise module for camera navigation exercise in gynaecology (right)

ET is designed following a modular approach, in which the anatomical reproduction can be easily replaced to reproduce different scenarios, extending exercises portfolio and its usability in different endoscopic surgical specialties. Figure 1 left shows the main components of the system.

The main module contains all required hardware and software necessary for the execution, measurement and evaluation of the exercises: an embedded PC, a touch screen acting as interactive graphical interface, a Tool Tracker Arm (TTA) to measure the endoscope position, a high-resolution camera attachable to the endoscope, a communication port with the exercises modules and, finally, a pedal to execute several actions during the exercises. The TTA is a passive arm that measures the position and orientation of the endoscope tip. The arm has 6DoF, configured as passive rotational joints. Due to the kinematic constrains introduced by the fulcrum point, situated at the entrance of the organ’s cavity, only four TTA’s joints, $(\Theta _1, \Theta _2, \Theta _3, \Theta _6)$, are equipped with rotational encoders. This configuration is sufficient to determine the position and orientation (POSE) of the tool tip. Figure 1 right shows the TTA kinematic configuration and the RCM pivoting point inside the exercise module.

Users are provided with a real endoscope and auxiliary tools (e.g. grasper for polyp extraction) attached to a camera using an optical coupler to allow endoscope rotation. A pedal is used to execute certain actions (e.g. indicate when a target is focused) to train the combined use of pedal and endoscope as in real clinical practice. During the execution of the exercises, the Graphical User Interface (GUI) shows the endoscopic images jointly with an interactive endoscopic tower. Users interact with this component to initialise the endoscopic system and resolve critical situations to improve the cognitive skills training and manage situation awareness in complex and realistic scenarios.

The Exercise Module (EM) contains the reproduction of the anatomy which is composed of the natural entrance orifice and the organ’s cavity (e.g. vagina and uterus or urethra and bladder). The geometry reproduces pre-defined standardised volumes and a realistic visual and haptic feedback using silicon-based materials with different additives. The cavities are equipped with the necessary sensors and actuators to measure user’s interaction (e.g. LEDs in targets to be focused, light sensors to detect polyp extraction, etc.).

### Exercises

The ET platform is currently equipped with two basic-skill training exercises based on GESEA Certification Programme developed by The Academy and ESGE. The exercises are named: Exercise 1: Camera Navigation (Level 1) and Exercise 2: Hand-eye Coordination (Level 2), which is out of the scope of this paper. Part of this program is based on the acquisition of practical skills in hysteroscopy using the HYSTT model, which is a test to measure the competence level in basic hysteroscopic psychomotor skills, [9, 27].

**Fig. 2:**
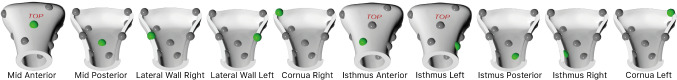
Illustration of the relevant anatomical positions inside the uterus

#### Exercise 1: 30-degree Camera Navigation

**Fig. 3:**
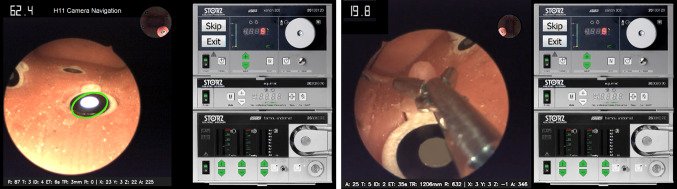
Screenshot of the 30-degree camera navigation exercise (left). Screenshot of the polyp extraction exercise (right)

Exercise 1 is oriented to train 30-degree endoscope navigation inside the organ’s cavity. Ten visual markers, in the form of a black circle encircling a round white LED, are arranged at relevant positions inside the uterine cavity, Fig. 2. Users should precisely focus each target within a maximum time limit. The order of the targets is randomised at each trial. The UI indicates the target to be focused (auditive and visual indications). The LED of the selected target is activated, and simultaneously, the GUI shows an ellipse that should be placed inside the surrounding target black ring. Users step on the pedal when they consider that the circle is correctly focused (all the ellipse is inside the black ring of the target). Figure 3 left shows a screen shot of the exercise execution.

The accuracy obtained at each target is computed as the percentage of ellipse inside the black ring when the user presses the pedal. The difficulty of the exercises can be modified in accordance with the learning progress: reducing maximum time to focus, changing the width of the ellipse to make the target focus more restrictive, not showing the anatomical position on the GUI and, finally adding more realistic visual conditions using AR and situation awareness. The analysis presented in this paper corresponds to a basic exercise without AR (only ellipses) and a maximum allowed time of 30 sec per target.

### Evaluation metrics and statistical tests

The study determines the proficiency of the subjects to navigate and locate relevant anatomical parts of the uterus. The measured parameters are the Execution time (Time to execute a trial), Accuracy (Mean of the accuracy obtained in reaching all the targets in each trial), Orientation error (Mean of the error between pre-defined endoscope orientation and orientation when the target is acquired), Economy of movement Trajectory Length (Tool tip trajectory length during a trial (in mm)), Economy of movement, Rotation (Total amount of endoscope rotation during a trial (in degree)).

Learning Curves (LC) along the 30 trials are also analysed to define the users evolution. To determine whether there are statistically significant differences for every evaluation criteria along LCs, the analysis studies the results obtained in the initial (1..3), middle (14..16) and final (28..30) trials. The study analyses Mean and Standard Deviation of every evaluation metrics, gains and differences between trial subsets, paired *t*-test, power test and, finally, sample size to determine the number of total users per subset.

### Experimental phase

The experimental phase was conducted in the gynaecological departments of the Hospital Sant Pau from Barcelona (Spain), the Consorci Sanitari de l’Anoia - Hospital d’Igualada (Spain) and in the head quarters of ESGE in Leuven (Belgium). The experiment sample is composed of a total of 80 gynaecologist with background in hysteroscopy but with different practical experience (from null to more than 200 hysteroscopies during the last 5 years). The experiment, executed in a single session per subject, was divided into three sequential phases: introduction, trials and survey.

**Fig. 4:**
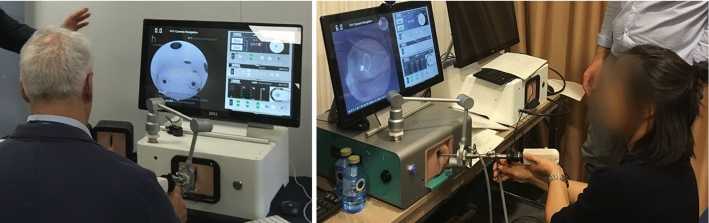
Subjects executing the camera navigation exercise

Five different target orders were defined to prevent the memory effect (users learn the order of the movements instead of the use of the endoscope and spatial navigation). None of the participants had previous experience with the presented device, and no previous training trials were allowed. The experiments were conducted in dry-lab conditions, Fig. 4.

## Results

### Statistical analysis

This section reviews the obtained results, analysing the obtained LC of all evaluation indices. Following a comparative study between initial, middle and final trials is performed to observe if there are statistical significant improvements along the trials.

**Fig. 5:**
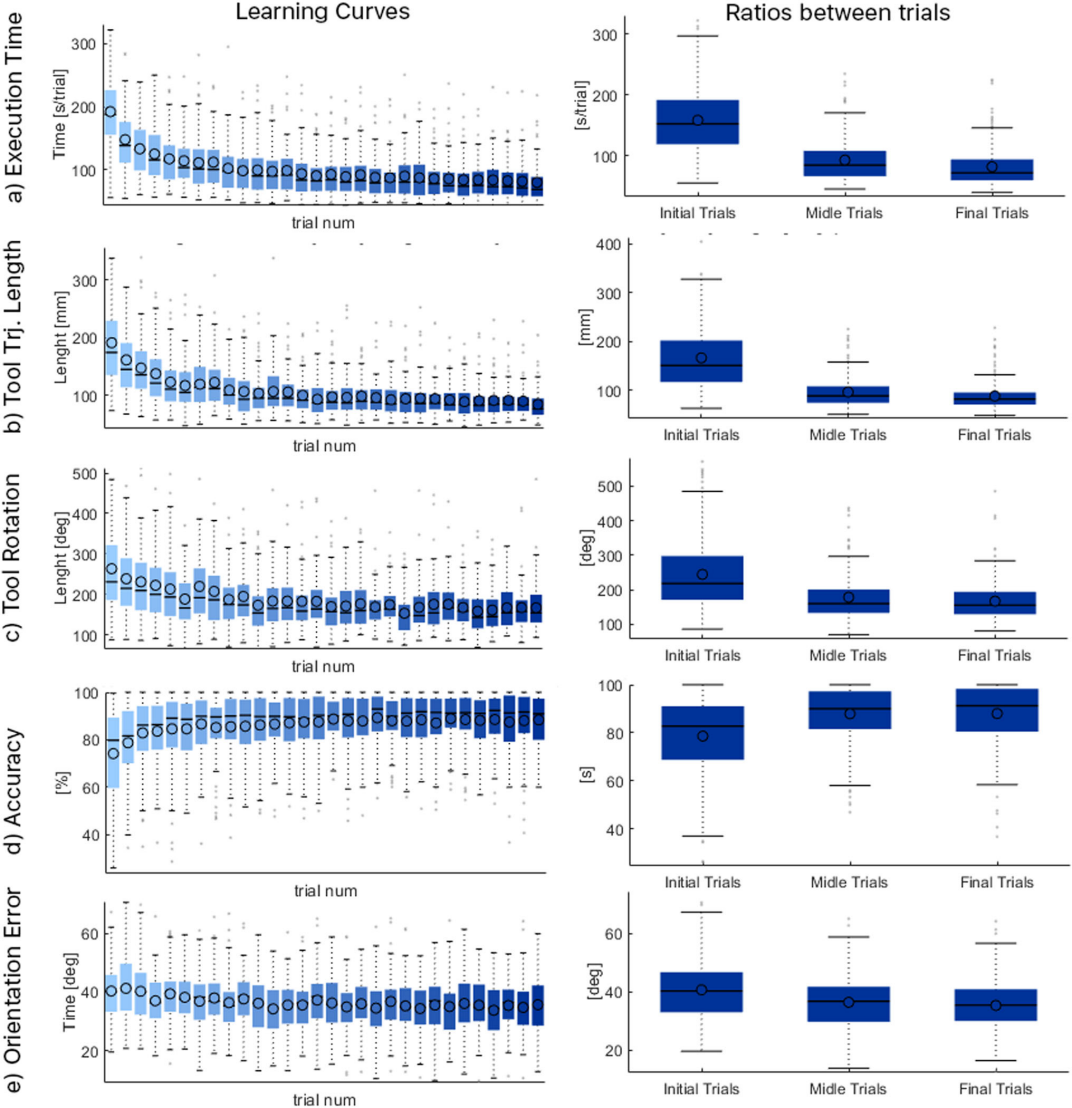
Statistics of all evaluation criteria along LC (left) and statistics of each subset (right)

The LCs points out that all users have obtained statistically significant improvements in all the studied criteria. More compact interquartiles indicate that the training benefits are valid for all type of users, independently from their previous experience. Obtained LC from Execution Time, Fig. 5a, and trajectories described by the endoscope (Trajectory Length, Fig. 5b and Tool Rotation, Fig. 5c) present logarithmic evolution of the trials means. Accuracy, Fig.5d, and Orientation Error, Fig. 5e, also present a noticeable improvement along their LCs. It is necessary to point out that the Orientation Error is the evaluation criteria that shows lower improvements and requires a new approximation, establishing a range of valid orientations defined by the target orientation instead of a single value for a more realistic evaluation (lateral targets should have more strict valid orientation ranges compared with cornuas, istmus and mid-targets).

**Fig. 6:**
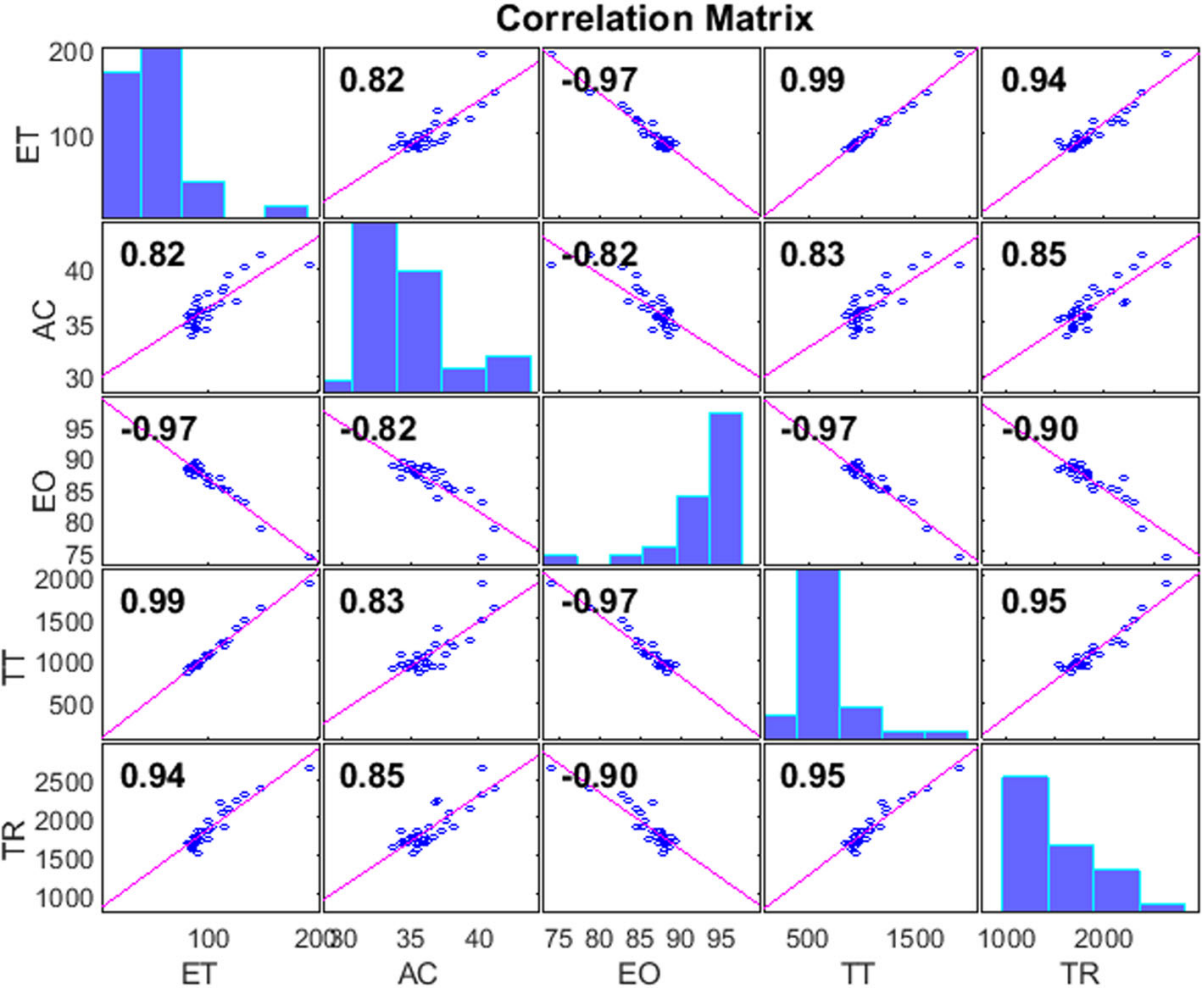
Correlation analysis between evaluation criteria. ET: Execution Time, AC: Accuracy, EO: Error of Orientation, TT: Trajectory Translation, TR: Trajectory Rotation

The obtained LC from all evaluated aspects present high correlation values. The evolution of users tends towards a clear common improvement in all parameters: lower execution time, with shorter trajectories and the exploration, is more accurate. Figure 6 shows the Pearson’s linear correlation. The resulting $p<0.001$ for all paired correlations indicate maximum significance.

#### Initial vs final trials

All evaluation metrics, Table 1, present statistical significant improvements comparing initial and final trials, indicating the learning effect of the platform. The ratios of endoscope translation and rotation are particularly noteworthy: 1.844 and 1.906, respectively, indicating that users have learned its usage. The accuracy also increased, although to a lesser extent. However, if the combined factor of improved accuracy and time reduction (gain of 1.935) is considered, the results are much more remarkable. The combined improvement of all relevant aspects indicate a noticeable benefit of the training platform. In addition, the dispersion of the results decreases at the final trials compared with the initial ones, indicating that the obtained benefits are valid for all users, regardless of their experience.

**Table 1:** Statistics for exercise 1. Comparison between initial and final trial subsets

	meanIni	stdIni	meanEnd	stdEnd	*Ini*/*End*	${Ini-End}$	ttest				Power		Sample size	
							h	*p*	$ci_{Inf}$	$ci_{Sup}$	Ini	End	Ini	End
Exec time	157,984	55,753	81,648	32,514	1.935	76,336	1.000	0.000	68,145	84,527	1.000	1.000	10	5
Accuracy	78,553	16,918	87,965	12,329	0.893	−9.412	1.000	0.000	−12,068	−6.756	1.000	1.000	44	4
err orientation	40,591	10,650	35,263	9140	1.151	5.328	1.000	0.000	3.548	7.108	1.000	1.000	54	4
Tool translation	1,666,479	712,278	887,263	285,724	1.878	779,217	1.000	0.000	681,747	876,686	1.000	1.000	13	6
Tool rotation	2443,708	1057,438	1669,713	550,910	1.464	773,996	1.000	0.000	622,638	925,354	1.000	1.000	27	7
Ratio tool translation	6548	3771	3551	1420	1.844	2997	1.000	0.000	2485	3508	1000	1.000	23	10
Ratio tool rotation	10,520	10,735	5520	5.112	1906	5000	1.000	0.000	3490	6510	1.000	1.000	62	54

#### Initial vs middle trials

The major gains in all evaluation indices are concentrated in this interval, Table 2, obtaining statistically significant differences. Particularly remarkable is the accuracy: all gain is obtained in this interval. All users generate shorten and straighten trajectories to the targets in the middle trials compared with the initial ones. Some users did not maintain the proper camera orientation (preserving the horizontal camera orientation) during initial trials. The result is a noticeable improvement in execution time, trajectory length and accuracy and a proper learning of the 30 degree endoscope use.

**Table 2:** Statistics for exercise 1. Comparison between initial and middle trial subsets

	meanIni	stdIni	meanEnd	stdEnd	*Ini*/*End*	${Ini-End}$	ttest				Power		Sample size	
							h	*p*	$ci_{Inf}$	$ci_{Sup}$	Ini	End	Ini	End
Exec time	157,984	55,753	92,586	35,927	1.706	65,398	1.000	0.000	56,982	73,815	1.000	1.000	12	6
Accuracy	78,553	16,918	87,885	11,334	0.894	−9.332	1.000	0.000	−11,916	−6748	1.000	1.000	45	4
err orientation	40,591	10,650	36,325	9420	1117	4266	1.000	0.000	2462	6069	1.000	1.000	83	4
Tool translation	1666,479	712,278	969,508	331,189	1.719	696,971	1.000	0.000	597,234	796,707	1.000	1.000	16	6
Tool rotation	2443,708	1057,438	1783,783	730,904	1.370	659,925	1,000	0,000	496,831	823,019	1,000	1,000	36	8
Ratio tool translation	6548	3771	4179	1534	1.567	2.369	1.000	0.000	1852	2886	1.000	1.000	35	10
Ratio tool rotation	10,520	10,735	6061	5542	1736	4459	1.000	0.000	2925	5993	1.000	1.000	78	63

**Table 3:** Statistics for exercise 1. Comparison between middle and final trial subsets

	meanIni	stdIni	meanEnd	stdEnd	*Ini/End*	${Ini-End}$	ttest				Power		Sample size	
							h	$\textit{p}$	$ci_{Inf}$	$ci_{Sup}$	Ini	End	Ini	End
Exec time	92,586	35,927	81,648	32,514	1.134	10,938	1.000	0.001	4792	17,083	0.997	1.000	143	7
Accuracy	87,885	11,334	87,965	12,329	0.999	$-$0.080	0.000	0.941	$-$2204	2044	0.051	1000	260811	3
err orientation	36,325	9420	35,263	9140	1.030	1.062	0.000	0.211	$-$0,602	2727	0.413	1000	1024	4
Tool translation	969,508	331,189	887,263	285,724	1.093	82,246	1000	0.004	26,764	137,728	0.969	1.000	213	6
Tool rotation	1783,783	730,904	1669,713	550,910	1.068	114,071	0.000	0.054	$-$2,041	230,183	0.673	1.000	536	7
Ratio tool translation	4179	1534	3551	1420	1177	0.628	1000	0.000	0.363	0.893	1.000	1.000	80	7
Ratio tool rotation	6061	5542	5520	5112	1.098	0.541	0.000	0.267	$-$0.415	1.498	0.325	0.752	1365	446

**Fig. 7:**
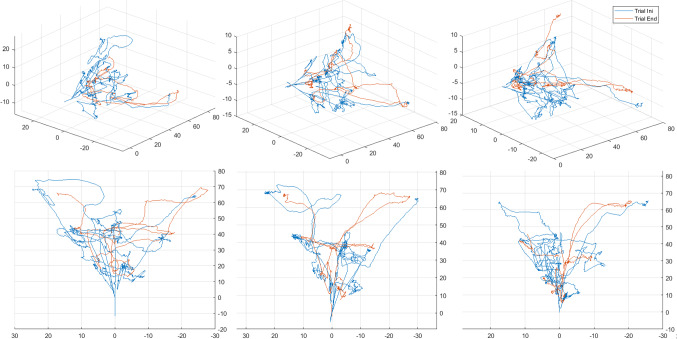
Three examples of endoscope trajectories at initial and final trials. The comparison evidences the improvements obtained during the trials

#### Middle vs final trials

Gains are significantly reduced compared to Ini-Mid. There are statistically significant differences only in Exec Time and Tool Translation. There is reduction of the standard deviation and more compact quartiles, indicating that the majority of subjects improved their proficiency at the end of all repetitions. Table  3 shows the statistical analysis results. A remarkable aspect of this phase is that the expected subjects’ fatigue has not been detected.

## Discussion

The experiments have demonstrated the benefits of the training platform. The multivariable analysis points out that all subjects have obtained statistically significant improvement in all relevant parameters, independently from their previous experience. In addition, the analysis of the Ini-Mid and Mid-End intervals indicates that the major learning effect concentrates in the initial part. The result is a hysteroscopy with shorter execution time combined with higher accuracy, shorter and straighten trajectories and a better use of the 30-degree endoscope to explore the anatomy of the internal uterus walls. Figure 7 shows several examples of initial and final trajectories carried out by different subjects (the selection has been randomly generated). In them, it is possible to observe how the subjects generate trajectories with more defined patterns, reaching the targets in a more direct form as they advance in training. During fine target focusing, an interesting difference appears: while in the first attempts small movements are observed to focus the target, in the last attempts they disappear.

The positive results encourage to evolve the platform to generate more exercises and extend to cystoscopy to explore the urinary bladder. The analysis of all relevant data opens the possibility of generating personalised curricula to accompany the learning phase of each subject in a personalised manner.

## Conclusions

ET exploits the advantages of physical (real tools, haptic feedback and environment interaction), and virtual (multi-parametric evaluation and AR) training platforms. The modular construction enables the use of ET in different medical specialties that use natural orifice entrance. The result is a highly realistic platform with objective evaluation and mentoring capabilities. The implementation and validation of the platform has started with the specialty of gynaecology. This paper has demonstrated the validity of the platform using the intra-uterine navigation with a 30-degree endoscope. The obtained learning curves indicate continuous improvement in all relevant parameters with a high correlation index between them. Future work will be oriented to identify differences between subjects based on previous experience to obtain platform construct and content validity.

## Supplementary Information

Below is the link to the electronic supplementary material.

Supplementary file 1 (mp4 28036 KB)

## Data Availability

Proprietary of SurgiTrainer.

## Declarations

### Code availability

Proprietary software.
